# Common and rare variants of *WNT16*, *DKK1* and *SOST* and their relationship with bone mineral density

**DOI:** 10.1038/s41598-018-29242-8

**Published:** 2018-07-19

**Authors:** Núria Martínez-Gil, Neus Roca-Ayats, Anna Monistrol-Mula, Natàlia García-Giralt, Adolfo Díez-Pérez, Xavier Nogués, Leonardo Mellibovsky, Daniel Grinberg, Susana Balcells

**Affiliations:** 1 Department of Genetics, Microbiology and Statistics, Faculty of Biology, University of Barcelona, IBUB, IRSJD, CIBERER, Barcelona, Spain; 2 Musculoskeletal Research Group, IMIM (Hospital del Mar Medical Research Institute), Centro de Investigación Biomédica en Red de Fragilidad y Envejecimiento Saludable (CIBERFES), ISCIII, Barcelona, Spain

## Abstract

Numerous GWAS and candidate gene studies have highlighted the role of the Wnt pathway in bone biology. Our objective has been to study in detail the allelic architecture of three Wnt pathway genes: *WNT16*, *DKK1* and *SOST*, in the context of osteoporosis. We have resequenced the coding and some regulatory regions of these three genes in two groups with extreme bone mineral density (BMD) (n = ∼50, each) from the BARCOS cohort. No interesting novel variants were identified. Thirteen predicted functional variants have been genotyped in the full cohort (n = 1490), and for ten of them (with MAF > 0.01), the association with BMD has been studied. We have found six variants nominally associated with BMD, of which 2 *WNT16* variants predicted to be eQTLs for *FAM3C* (rs55710688, in the Kozak sequence and rs142005327, within a putative enhancer) withstood multiple-testing correction. In addition, two rare variants in functional regions (rs190011371 in *WNT16b* 3′UTR and rs570754792 in the *SOST* TATA box) were found only present in three women each, all with BMD below the mean of the cohort. Our results reinforce the higher importance of regulatory versus coding variants in these Wnt pathway genes and open new ways for functional studies of the relevant variants.

## Introduction

In the last decade, numerous genome wide association studies (GWAS) have been conducted as a strategy to identify new genes and variants associated with complex diseases. In the field of osteoporosis, a number of GWAS have been performed to determine the association with bone mineral density (BMD) and osteoporotic fracture. BMD is a complex quantitative trait that is mostly genetically determined, therefore it is a good biological marker for the determination of bone status. Initial GWAS studies^1–6^ have identified more than 50 *loci* associated with BMD, among which the Wnt/βcatenin signaling pathway was especially enriched. Specifically, in the study by Estrada *et al*.^6^, 14 BMD-associated *loci* were also found to be associated with fracture risk, and 6 of them belonged to the Wnt pathway: *LRP5, SOST, DKK1, WNT4, WNT16* and *CTNNB1*. More recently a low-frequency variant with large effects on BMD has been described in the *WNT16* gene in a whole genome sequencing study^7^. In fact, several monogenic bone phenotypes are caused by rare mutations in genes of the Wnt pathway. For example, sclerosteosis^8–10^ and van Buchem^11,12^ – two monogenic bone overgrowth diseases- are caused by rare loss of function mutations or the deletion of an enhancer element (ECR5) of *SOST*, and the osteoporosis-pseudoglioma syndrome is caused by rare loss of function mutations of *LRP5*^13^.

The Wnt pathway regulates a wide variety of cellular processes during embryogenesis and in adult regenerative tissues, as is the case for bone. Wnt proteins can activate up to 3 signaling pathways: 2 non-canonical and one canonical pathway or β-catenin-dependent pathway. When this signaling pathway is activated in osteoblasts, a series of important genes for the differentiation and formation of bone are induced^14^. The canonical pathway is finely regulated by extracellular inhibitors, including DKK1 and sclerostin, encoded by *DKK1* and *SOST*, respectively. Sclerostin is produced mainly in osteocytes, although it is also expressed in other cell types differentiated from the mineralized matrix, such as cementocytes or chondrocytes^15^.

Despite the large number of *loci* associated with BMD, they account for only a small part of the genetic impact on the femoral neck BMD (<6%)^6^. Thus, the variability of BMD cannot be explained solely by tag SNPs, which is why a search for new polymorphisms and rare variants is necessary. Our objective has been to identify variants within 3 genes of this pathway: *WNT16*, *DKK1* and *SOST*. We have approached the characterization following a two-step process. First, we have resequenced the coding regions of these genes in two extreme BMD groups of postmenopausal women of the BARCOS cohort to look for rare variants responsible for the phenotype, or common variants associated with BMD. Secondly, we analyzed the association of the common and low frequency variants with BMD, in the full cohort, and we explored the distribution of the rare variants.

## Results

### Search for Variants in a Truncated Selection of Samples from the BARCOS Cohort and Analysis of their Frequencies in the Extreme Groups

**Figure 1:**
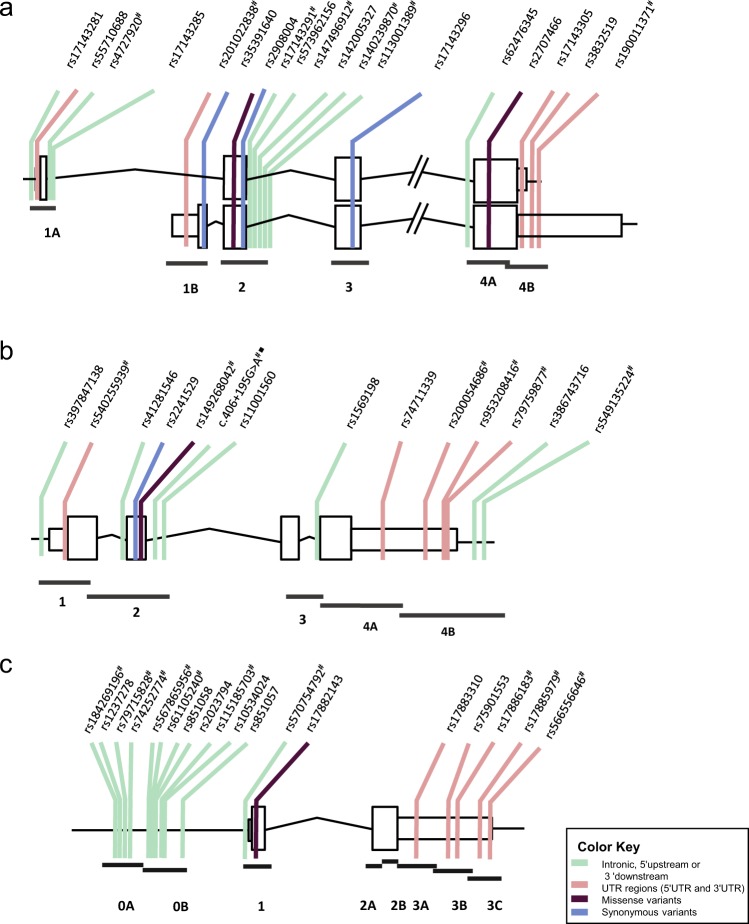
Variants found in *WNT16* (**a**), *DKK1* (**b**) and *SOST* (**c**). Horizontal lines below the genes indicate amplicons used to resequence the exons and flanking intronic regions (not to scale). ^#^Rare variants (MAF < 0.01). ^■^Novel variant.

We resequenced *WNT16*, *DKK1* and *SOST* coding exons, intronic flanks, UTRs and regulatory regions, in 108 postmenopausal women in the extremes of the BMD distribution of the BARCOS cohort (55 with HBM and 53 with LBM, see Methods). A total of 19, 14 and 18 single nucleotide variants (SNVs) were identified in *WNT16*, *DKK1* and *SOST*, respectively (Tables 1–3 and Fig. 1), of which only the *DKK1* variant c.406 + 195 G > A was novel. Fifteen of them have frequencies above 5% (common variants), 12 have frequencies between 1–5% (low frequency variants, LFV), and the 24 remaining have frequencies below 1% (rare variants), according to EUR population in 1000 genomes (sequencing chromatograms of the 24 rare variants are shown in Supplementary Figures S1–3). Only the *DKK1* LFV rs74711339 showed significant differences between the genotype frequencies of the two extreme groups (Fisher’s exact test, *p* = 0.0224). This SNP is located in the 3′UTR and the minor allele (G) was found overrepresented in the LBM group (Table 2).

**Table 1:** Variants found in the resequencing of *WNT16*.

Variant definition			rs Number	Type		MAF (EUR)	Number of alleles		Functional prediction
*WNT16* (genomic)	*WNT16a*	*WNT16b*		*WNT16a*	*WNT16b*		HBM	LBM	
g.120965352 C > T	g.120965352 C > T	g.120965352 C > T	rs17143281	5′UP	5′UP	0.036 (T)	7	3	eQTL
g.120965467_120965470dupCCCA	p.Met1?	g.120965467_120965470dupCCCA	rs55710688	Fs	5′UP	0.233 (CCCA)	31	23	eQTL
g.120965562 A > G	c.65 + 28 A > G	g.120965562 A > G	rs4727920	I	5′UP	0.003 (G)	3	1	
g.120965585 T > A	c.65 + 51 T > A	g.120965585 T > A	rs17143285	I	5′UP	0.041 (A)	3	7	
g.120969332 C > A	c.66–289 C > A	c.−15C > A	rs201022838	I	5′UTR	0 (A)	1	0	
g.120969412 G > C	c.66–209 G > C	p.Leu22Leu	rs35391640	I	S	0.038 (C)	4	2	
g.120969769 G > A	p.Gly72Arg	p.Gly82Arg	**rs2908004**	M	M	0.44 (A)	51	38	eQTL; T/B/P
g.120969825 C > A	p.Thr90Thr	p.Thr100Thr	rs17143291	S	S	0.009 (A)	0	2	
g.120969922_120969924delCTC	c.316 + 51_316 + 53delCTC	c.346 + 51_346 + 53delCTC	rs573962156	I	I	0.015 (-)	1	2	
g.120969929 G > T	c.316 + 58 G > T	c.346 + 58 G > T	rs147496912	I	I	0.009(T)	1	1	
g.120969974_120969975dupCT	c.316 + 103_316 + 104dupCT	c.346 + 103_346 + 104dupCT	**rs142005327**	I	I	0.254 (CT)	30	23	eQTL; T/B/P
g.120970018 A > G	c.316 + 147 A > G	c.346 + 147 A > G	rs140239870	I	I	0.006 (G)	0	2	
g.120970045 C > A	c.316 + 174 C > A	c.346 + 174 C > A	*rs113001389*	I	I	0 (A)	1	0	
g.120972015 G > A	p.Arg200Arg	p.Arg210Arg	rs17143296	S	S	0.012 (A)	1	2	
g.120978801 T > C	c.604_134T > C	c.634_134T > C	rs62476345	I	I	0.018 (C)	3	0	
g.120979089 C > T	p.Thr253Ile	p.Thr263Ile	**rs2707466**	M	M	0.44 (T)	51	38	eQTL
g.120979512 C > T	c.*113 C > T	c.*113 C > T	rs17143305	3′UTR	3′UTR	0.162 (T)	16	11	miRNA; eQTL
g.120979527_120979528insCTCT	g.120979527_120979528insCTCT	c.*128_*129insCTCT	rs3832519	3′ D	3′UTR	0.177 (CTCT)	19	16	
g.120979568 G > C	g.120979568 G > C	c.*169 G > C	*rs190011371*	3′ D	3′UTR	0 (C)	0	1	miRNA

Fs: Frameshift; I: Intronic; M: Missense; S: Synonymous; 5′UP: 5′upstream; 3′D: 3′ downstream. In bold, SNPs genotyped in the complete BARCOS cohort. In italics, rare variants genotyped in the complete BARCOS cohort. MAF (EUR) from 1000 genomes.

miRNA: Variant that affect the binding of miRNA (see Supplementary Table S1); eQTL: Variant described in GTEx as an eQTL in different tissues (see Supplementary Table S2).

T: Tolerated by SIFT; B: Benign by PolyPhen-2; P: Polymorphism by Mutation Taster.

**Table 2:** Variants found in the resequencing of *DKK1*.

Variant definition		rs Number	Type	MAF (EUR)	Number of alleles		Functional prediction
*DKK1*					HBM	LBM	
g.54073904dupC	g.54073904dupC	rs3456455	5′UP	0.012 (C)	3	5	
g.54074079 C > A	c.−116C > A	rs540255939	5′UTR	0.001 (A)	0	1	
g.57074660 C > T	c.244-23 C > T	rs41281546	I	0.056 (T)	8	12	eQTL
g.54074757 A > G	p.Ala106Ala	rs2241529	S	0.458 (A)	36	39	
g.54074798 G > T	p.Arg120Leu	rs149268042	M	0.008(T)	1	0	D/PD/DC
g.54075040 G > A	c.406 + 195 G > A	—	I	—	1	0	
g.54075127 T > C	c.406 + 282 T > C	rs11001560	I	0.445 (T)	28	39	
g.54076271 A > G	c.548-43 A > G	**rs1569198**	I	0.51 (A)	45	52	eQTL; Splicing
g.54076649 A > G	c.*82 A > G	**rs74711339**	3′UTR	0.043 (G)	5	11	eQTL
g.54076944delT	c.*377delT	rs200054686	3′UTR	0 (-)	0	1	
g.54077319 C > T	c.*752 C > T	rs953208416	3′UTR	—	1	0	
g.54077322 G > A	c.*755 G > A	rs79759877	3′UTR	0 (A)	1	0	
g.54077566_54077571delCAGTATinsTAA	g.54077566_54077571delCAGTATinsTAA	rs386743716	3′ D	—	8	13	
g.54077585 G > A	g.54077585 G > A	rs549135224	3′ D	0.001 (A)	2	0	

I: Intronic; M: Missense; S: Synonymous; 5′UP: 5′upstream; 3′D: 3′ downstream. In bold, SNPs genotyped in the complete BARCOS cohort. MAF (EUR) from 1000 genomes.

eQTL: Variant described in GTEx as an eQTL in different tissues (see Supplementary Table S2); Splicing:Predicted to affect splicing (see Text).

D: Deleterious by SIFT; PD: Probable Damaging by PolyPhen-2; DC: Disease Causing by Mutation Taster.

**Table 3:** Variants found in the resequencing of *SOST*.

Variant definition		rs Number	Type	MAF (EUR)	Number of alleles		Functional prediction
*SOST*					HBM	LBM	
g.41838340 G > A	g.41838340 G > A	rs184269196	5′UP	0.002 (A)	2	1	
g.41838229 C > T	g.41838229 C > T	**rs1237278**	5′UP	0.355 (C)	45	40	eQTL
g.41838130 G > A	g.41838130 G > A	rs79715828	5′UP	0.005 (A)	1	0	
g.41838012 G > T	g.41838012 G > T	rs74252774	5′UP	0.002 (T)	1	0	
g.41837786 C > T	g.41837786 C > T	rs567865956	5′UP	0.003 (T)	2	1	
g.41837720 C > T	g.41837720 C > T	rs61105240	5′UP	0 (T)	1	0	
g.41837719 G > A	g.41837719 G > A	rs851058	5′UP	0.402 (A)	47	42	eQTL
g.41837660 T > C	g.41837660 T > C	**rs2023794**	5′UP	0.044 (C)	6	3	eQTL
g.41837530 C > A	g.41837530 C > A	rs115185703	5′UP	0 (A)	1	0	
g.41837510_41837512delTCC	g.41837510_41837512delTCC	rs10534024	5′UP	0.354 (TCC)	45	43	
g.41837264 G > C	g.41837264 G > C	rs851057	5′UP	0.123 (G)	12	14	
g.41836179 G > A	g.41836179 G > A	*rs570754792*	5′ UP	0.002 (A)	0	1	
g.41836082 C > T	p.Val10Ile	**rs17882143**	M	0.018 (T)	4	4	T/B/P
g.41832390 G > A	c.*320 C > T	**rs17883310**	3′UTR	0.013(T)	3	1	miRNA
g.41831844 G > A	c.*866 C > T	rs75901553	3′UTR	0.069 (T)	8	9	miRNA
g.41831706 C > T	c.*1004 G > A	rs17886183	3′UTR	0.004 (A)	4	1	miRNA
g.41831307delC	c.*1404delG	rs17885979	3′UTR	0.001 (-)	1	0	
g.41831153 C > G	c.*1557 G > C	rs566556646	3′UTR	0.001 (G)	1	0	

M: Missense; 5′UP: 5′upstream. In bold, SNPs genotyped in the complete BARCOS cohort. In italics, rare variants genotyped in the complete BARCOS cohort. MAF (EUR) from 1000 genomes.

miRNA: Variant that affect the binding of miRNA (see Supplementary Table S1); eQTL: Variant described in GTEx as an eQTL in different tissues (see Supplementary Table S2).

T: Tolerated by SIFT; B: Benign by PolyPhen-2; P: Polymorphism by Mutation Taster.

### *In silico* Analysis of Functionality of the Identified Variants

To better assess the importance of the identified variants we have explored their possible functionality using *in silico* tools and databases.

Four of the variants were missense, of which 2 common SNPs in *WNT16* (rs2908004; p.Gly72Arg/p.Gly82Arg and rs2707466; p.Thr253Ile/p.Thr263Ile) and one LFV in *SOST* (rs17882143; p.Val10Ile) were predicted to be tolerated and benign by SIFT and PolyPhen-2, respectively. The other missense change, the *DKK1* rare variant rs149268042 (p.Arg120Leu), was predicted to be harmful (deleterious by SIFT, probably damaging by PolyPhen-2, and disease causing by Mutation Taster).

Fifteen other variants might play some regulatory role. One of them, the rs55710688 SNP, located in the 5′UTR of *WNT16a*, is an insertion of 4 nt (CCCA) that affects the Kozak consensus sequence, and might alter translation initiation rate. The *DKK1* intron 3 SNP rs1569198 was predicted to affect splicing, where the G allele might generate a new splice acceptor site 42 nt upstream of the original one, although with a lower score. One rare variant in *SOST*, rs570754792, located 23 bp upstream the transcription start site, lies within the extended TATA box motif. The change is a C (most probable in the consensus TATA box sequence) for a T (least probable). Five 3′UTR variants were predicted to have an effect on the binding of 13 miRNAs (Supplementary Table S1): 3 SNPs in *SOST* (rs17883310, rs17886183, rs75901553), 1 SNP in *WNT16* (rs17143305) and 1 rare variant in *WNT16b* (rs190011371), not present in the other mature transcript of this gene. Several variants were shown to be eQTLs of *WNT16, DKK1* or *SOST*, according to GTEx. The *WNT16* SNP rs55710688 that affects the Kozak sequence mentioned above, was also described as an eQTL of *WNT16* in adipose tissue. Regarding *DKK1*, two SNPs (rs74711339 and rs41281546) are eQTLs in transformed fibroblasts. For the *SOST* gene, we have found 3 SNPs (rs1237278, rs851058 and rs10534024) that act as eQTLs in more than one tissue. This information together with additional eQTL information relative to other genes, is shown in Supplementary Table S2. Finally, the *WNT16* SNP rs142005327 (a 2-bp intronic insertion) and the rare variant rs113001389 lie in a potential regulatory site in osteoblasts, according to ENCODE data (and rs142005327 is described as an eQTL of *CPED1* and *FAM3C*).

### Association of Common and Low Frequency Variants with BMD in the Complete Cohort

**Figure 2:**
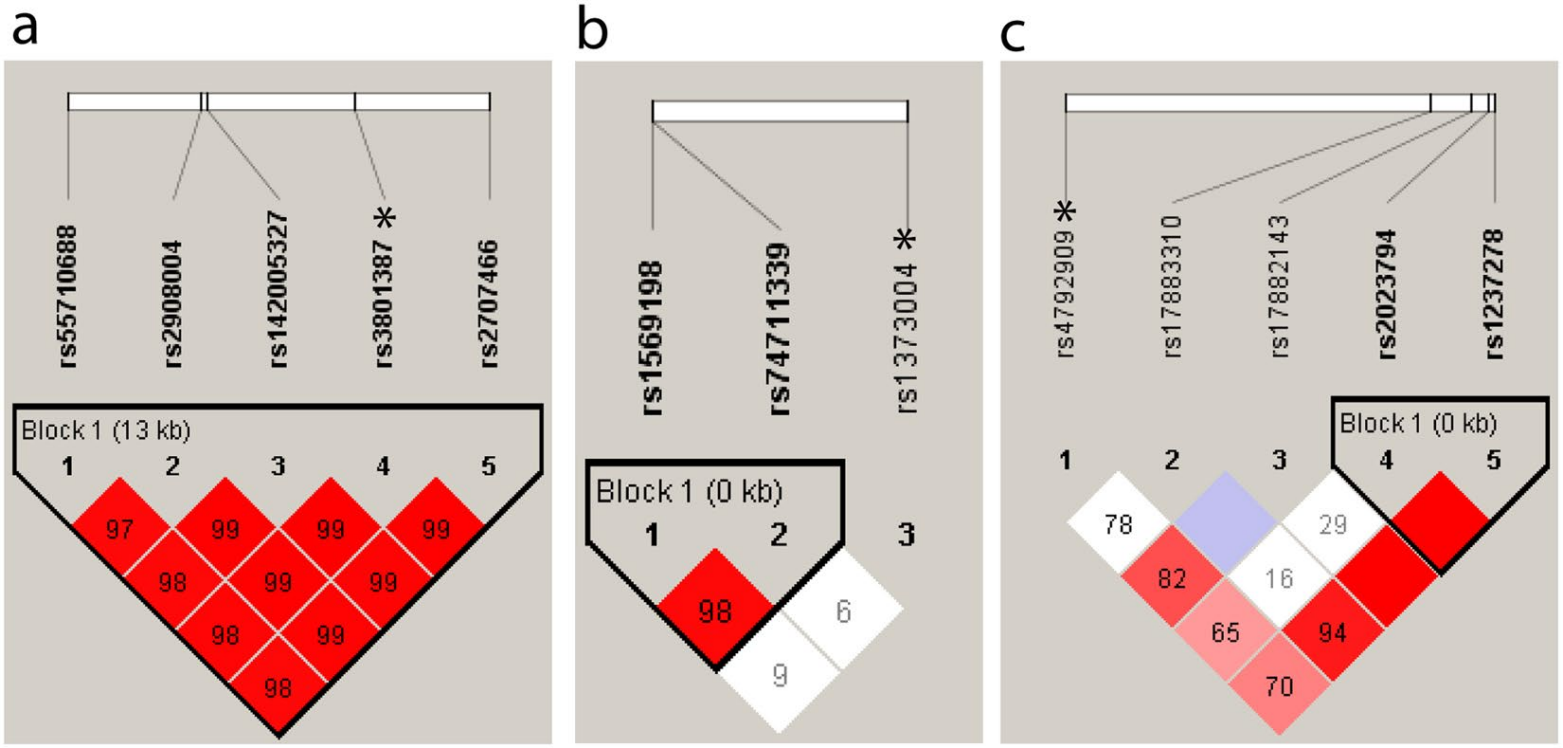
Haploview linkage disequilibrium plots of variants of *WNT16* (**a**), *DKK1* (**b**) and *SOST* (**c**) genotyped in the BARCOS cohort. *GWAS hits from Estrada *et al*.^6^. The numbers within the squares and the color scale both refer to D’/LOD values (with bright red: D’ = 1 and LOD ≥ 2; white: D’ < 1 and LOD < 2; blue: D’ = 1 and LOD < 2 and shades of pink/red: D’ < 1 and LOD ≥ 2).

In total, six common variants and four low frequency variants were genotyped in the complete BARCOS cohort (n = 1490) to test their association with BMD (Tables 1–3, in bold). For all of them, we obtained MAF values that are similar to those found in 1000 genomes for the European population (Supplementary Table S3). Nominal significant differences were obtained for lumbar spine BMD (LS-BMD) with the *WNT16* SNPs rs55710688, rs142005327, rs2908004 and rs2707466, under an additive model, and for the *SOST* SNP rs17882143, under a dominant model (Table 4, italics). In all cases, the minor allele had a protective effect on LS-BMD (Supplementary Figure S4). Moreover, nominal significant differences were obtained in the comparison of the femoral neck BMD (FN-BMD) with the *WNT16* SNPs rs55710688, rs142005327 and rs2707466 and for the *DKK1* SNP rs1569198, under an additive model. Only the *WNT16* SNPs rs55710688 and rs142005327 remained significant after Bonferroni’s correction (p < 0.0038) (Table 4, bold). No significant association was found between the genotyped variants and fractures. The *SOST* SNP rs17882143 presented the largest effect size (β = 0.041) of all associated SNPs described (Supplementary Table S4). In our cohort, the associated SNPs in *WNT16* and *SOST* were found to be in linkage disequilibrium with the respective GWA hits (rs3801387, *WNT16*; rs4792909, *SOST*) of Estrada *et al*.^6^ (Fig. 2a,c), while the *DKK1* SNP rs1569198 was not (Fig. 2b).

**Table 4:** Association between LS-BMD and FN-BMD and genotypes of common and LFV *WNT16*, *DKK1* and *SOST* variants.

SNP	Position	Type	*P* value LS-BMD			*P* value FN-BMD		
			A	R	D	A	R	D
*WNT16*								
rs55710688	p.Met1? g.120965467_120965470dupCCCA	Fs 5′UP	***0.0037***	*0.0283*	*0.0116*	*0.0479*	0.1026	0.0991
rs2908004	p.G72R/p.G82R	M	*0.0263*	0.2152	*0.0192*	0.0510	0.0894	0.1254
rs142005327	c.346 + 103_104dupCT c.316 + 103_104dupCT	I	***0.0037***	*0.008*	*0.0235*	*0.0191*	0.1454	*0.0285*
rs2707466	p.T253I/p.T263I	M	*0.0182*	0.1252	*0.0188*	*0.0167*	0.0504	*0.0439*
rs3801387^#^	c.603 + 2747 A > G c.633 + 2747 A > G	I	*0.0081*	*0.0060*	0.0609	*0.0416*	0.1032	0.0862
*DKK1*								
rs1569198	c.548–43 A > G	I	0.3626	0.726	0.2635	*0.0095*	*0.0362*	*0.0307*
rs74711339	c.*82 A > G	3′UTR	0.7960	0.0752	0.9498	0.0682	0.3901	0.0801
rs1373004^#^	10:g.54427825 T > G	I	0.0543	0.1582	0.0867	0.585	0.4641	0.4006
*SOST*								
rs1237278	g.41838229 C > T	5′UP	0.9395	0.7643	0.9395	0.0955	0.4533	0.0718
rs2023794	g.41837660 T > C	5′UP	0.5142	0.4199	0.6018	0.6703	0.6838	0.5965
rs17882143	p.Val10Ile	M	0.0669	0.1204	*0.0352*	0.9888		
rs17883310	c.*320 C > T	3′UTR	0.2236	0.6254	0.3454	0.6152	0.7935	0.6319
rs4792909^#^	g.41798824 G > T	5′UP	0.2158	0.1895	0.4355	0.1757	0.1537	0.3874

Fs: Frameshift; I: Intronic; M: Missense; 5′UP: 5′upstream; 3′D: 3′ downstream. Values in italics indicate nominal significance. Values in bold indicate Bonferroni’s significance. ^#^SNP previously genotyped in BARCOS (Estrada *et al*.)^6^. A: Additive model; R: Recessive model; D: Dominant model.

Reference allele for the dominant and recessive models was the minor allele.

### Haplotype analyses of *WNT16* associated SNPs

We next tested whether haplotypes might display a stronger association with BMD than any given *WNT16* SNP alone. We built haplotypes including the 4 associated SNPs and the *WNT16* tag-SNP from Estrada *et al*.^6^ in the complete BARCOS cohort and could detect 4 different haplotypes with frequencies above 2% (Table 5). The two most frequent ones consisted on the combination of all the major alleles or all the minor alleles, as expected, given the strong LD present in the region. We tested association between each of the 4 major haplotypes and LS-BMD and found that only the two most frequent (DGDAC and IAIGT) were nominally significantly associated (Table 5), although they did not reach significance after Bonferroni’s correction. DGDAC had a BMD-lowering effect, opposite to IAIGT, which was protective. These effects are in agreement with those of the individual SNPs. We could not find an association of any haplotype with FN-BMD (data not shown).

**Table 5:** Haplotype association with LS-BMD.

Haplotype	Freq	pValue	β
DGDAC	0.5479	*0.02743*	−0.01245
IAIGT	0.2586	*0.005418*	0.01815
DADAT	0.1522	0.9496	−0.00051
DAIGT	0.0212	0.5045	−0.01368

Haplotype: rs55710688, rs2908004, rs142005327, rs3801387 and rs2707466.

Nominal significant values are in italics.

We also tested for significant frequency differences of the 4 major haplotypes between the HBM and LBM groups of the truncate selection, and found none. We only observed a slight overrepresentation of DGDAC in LBM and a slight overrepresentation of IAIGT in the HBM group, as expected (Table 6). Finally, DGDAC was found to be significantly overrepresented in the lower half of the LS-BMD distribution in the complete BARCOS cohort (Table 6).

**Table 6:** Haplotype distribution in extreme BMD groups, and in the two halves of the complete BARCOS cohort according to LS-BMD mean (upper half > 0.8509; lower half < 0.8509).

Haplotype	Extreme BMD		Complete BARCOS cohort	
	HBM (n = 110)	LBM (n = 106)	Upper half (n = 1159)	Lower half (n = 1295)
DGDAC	60	67	653	748
IAIGT	30	21	345	310
DADAT	18	16	183	220
DAIGT	0	1	20	29
p-value^a^	0.351		*0.012*	
p-value^b^	0.1865		***0.0022***	

^a^p-value for the 2 × 4 contingency table.

^b^p-value for the 2 × 2 contingency table including only the 2 associated haplotypes.

Nominal significant values are in italic, Bonferroni’s significant values are in bold.

### Analysis of Rare and Low Frequency Variants

In the resequencing of *WNT16*, *DKK1* and *SOST* in the extreme BMD groups, we found 24 rare and 12 low frequency variants. In *WNT16*, the minor alleles of these variants were distributed between 23 HBM and 22 LBM women. In *DKK1*, they were found in 8 HBM and 6 LBM women. Finally, in *SOST*, they were distributed in 16 HBM and 9 LBM women. However, this difference did not reach significance.

Three rare variants found in two LBM and one HBM women and located in putative regulatory elements (two in *WNT16* and one in *SOST*), were genotyped in the complete BARCOS cohort (in italics in Tables 1 and 3, respectively). For the LBM variants (rs190011371 and rs570754792), the rare allele was found in heterozygosis in 3 women whose BMD was below the mean of the BARCOS cohort (0.737 g/cm^2^ and 0.7610 g/cm^2^ vs. 0.8509 g/cm^2^). For the HBM variant rs113001389 (*WNT16*), no other instance of it was found in the complete BARCOS cohort.

## Discussion

*WNT16*, *DKK1* and *SOST* have been previously associated with osteoporosis^6^. In this study, we have investigated variants within these genes as possible causes of the association. Specifically, we resequenced them in a truncate selection of women of the BARCOS cohort based on their Z-score. We found 19, 14 and 18 variants, respectively, 15 of which were common, 12 low frequency and 24 rare variants. Ten variants, with *in-silico* predicted functionality, were tested for association in the complete BARCOS cohort, and 6 of them (4 in *WNT16*, one in *SOST* and one in *DKK1*) gave nominal significant results with LS-BMD, FN-BMD or both. After correction for multiple testing, only two *WNT16* variants (one affecting the Kozak sequence and the other a putative intronic enhancer) remained significant. Finally, 2 rare variants present in putative regulatory sites (TATA box of *SOST* and 3′UTR of *WNT16b*) were each found in only 3 women of the BARCOS cohort, all 6 with BMD values below the cohort average.

### Association with BMD

Of the 4 *WNT16* variants nominally associated with BMD, rs2707466 and rs2908004 were missense. These results agree with those published by others in different cohorts using several bone parameters^16–21^. Although these missense variants were predicted to be tolerated and benign by SIFT and PolyPhen-2, respectively, rs2707466 is a change of a threonine to an isoleucine that directly abolishes a phosphorylation site, which causes a deleterious effect on the WNT16 protein^18^, while rs2908004 is a change from a glycine to an arginine, two amino acids with very different biochemical properties. These functional changes might somehow contribute to the association.

The other two *WNT16* variants nominally associated with BMD remained so after multiple-testing correction and thus, deserve further attention. One, rs55710688, modifies the Kozak sequence of *WNT16a*, and has been shown to produce changes in the translation of this transcript^21^. The transcription of *WNT16* generates two RNA isoforms, *WNT16a* and *WNT16b*, which differ in the first exon and in the 3′UTR region and are independently controlled by two alternative promoters^22^. The expression of the *WNT16a* is restricted to the pancreas, whereas *WNT16b* is expressed in multiple organs^22^, although bone expression data are not available. It would therefore be interesting to know which isoform is being expressed in bone. In the work of Hendrickx *et al*.^21^, they carried out *in vitro* translation studies and showed a higher translation efficiency for the minor allele (insCCCA). Also, this allele was found over-represented in the HBM group, similar to our results. Interestingly, the rs55710688 variant is listed as an eQTL for *WNT16* (subcutaneous adipose tissue) and also for *FAM3C* (skin). The other variant, rs142005327, is an insertion of 2 nucleotides in intron 2, which lies in a potential regulatory site in osteoblasts, and is an eQTL of *FAM3C* (skin and tibial nerve) and of *CPED1* (tibial artery). Consistent with our results, Hendrickx *et al*.^21^ found differences between genotype frequencies of this variant in two extreme BMD groups, where the minor allele (insCT) was over-represented in the HBM group. Overall, our results for these two variants are in the same line of those of Hendrickx *et al*.^21^, and we describe, for the first time, a significant association in a full cohort, reinforcing their importance as determinants of bone mass.

The 4 variants of *WNT16* described above are in high linkage disequilibrium with the GWAS hit from Estrada *et al*.^6^, rs3801387, which lies in the last intron of the gene. We note that this variant is also an eQTL of *FAM3C* (skin). Given its location next to *WNT16*, there has been a debate on the role of *FAM3C* in BMD determination (for example see ref.^16^). This gene encodes a citokine-like factor involved in epithelial to mesenchymal transition and recently has been shown to modulate osteogenic differentiation through Runx2 downregulation^23^. It is tempting to speculate that the biological effect of these 3 significantly associated variants of *WNT16* happens in part through their effects on *FAM3C* expression.

The *DKK1* rs1569198, found nominally associated with FN-BMD, is predicted to affect splicing. This same variant was found associated with hip axis length (HAL) by Piters *et al*.^24^, in young Caucasian men. However, Koromila *et al*.^25^ failed to find association with BMD or bone turnover markers in Greek postmenopausal women. This SNP is not in LD with the GWA hit (rs1373004, Estrada *et al*.^6^), which lies 3.5 kb downstream of the gene, and therefore may constitute an independent signal. On the other hand, the DKK1 LFV rs74711339, that showed significant differences in the two extreme groups and is a *DKK1* eQTL, showed a trend for association with FN-BMD, but did not reach significance in the full cohort, probably due to lack of power.

The only *SOST* variant found nominally associated with BMD was rs17882143 (p.Val10Ile), a missense change affecting the signal peptide of the sclerostin protein. This change may alter the cellular localization of the protein as shown for another signal peptide missense variant (p.Val21Leu) by Kim *et al*.^26^ To our knowledge, this is the first time that the rs17882143 SNP is found associated with BMD. It is in linkage disequilibrium with the *SOST* GWAS hit of Estrada *et al*.^6^, rs4792909, which, interestingly, did not show association in our cohort (see Table 4). We did not obtain association with BMD for the rest of the *SOST* SNPs tested, including rs2023794, which Zhang *et al*.^27^ had found associated with LS-BMD in postmenopausal Chinese women.

### Interesting rare variants

The *WNT16b* 3′UTR rs190011371, found in one LBM woman, was genotyped in the full cohort and two other cases presented with the minor allele, both with LS-BMD values below the mean of the cohort. According to *in silico* predictors, only the major allele (G) would bind the miRNA hsa-miR-383. Interestingly, this miRNA was found significantly under-expressed in osteoporotic bone (our unpublished results; see Supplementary Table S1). It is tempting to speculate that the inability of this miRNA to bind to the mutated 3′UTR from *WNT16b* would have a similar biological outcome, and this would be in agreement with the presence of the variant in 3 women with LS-BMD below the mean. Similarly, the *SOST* variant rs570754792, located within the extended TATA box motif, was found in one LBM woman, and was also present in two other women of the full cohort with a BMD below the mean. It should be noted that when rare and low frequency variants of each gene were taken into account, only those of *SOST* presented an unbalanced distribution, with an over-representation in the HBM extreme group. If we assume that most of them might be damaging, this would agree with the loss of the inhibitory activity of sclerostin in HBM women.

### Additional variants

Beside the 13 variants chosen to be genotyped in the complete BARCOS cohort due to the evidence of functionally, there are other variants which deserve discussion. Among them, we highlight the SNPs rs17143305 and rs75901553 in the 3′UTR region of the *WNT16* and *SOST* genes, respectively. According to various functional predictors, rs17143305 may affect the binding of miRNAs hsa-miR-541 and hsa-miR-4263 and has been reported as an eQTL for genes *RP11-3L10.1* and *FAM3C* in different tissues. Interestingly, hsa-miR-541 has been described as a negative regulator of osteoblastic differentiation^28^, in the only study in which it is studied in relation to bone tissues, to the best of our knowledge. On the other hand, as explained above, *FAM3C* was reported to have a negative effect on osteogenic differentiation^23^. Regarding rs75901553, different functional predictors suggest that the binding of up to 8 miRNAs might be affected, two of them being hsa-miR-98-5p and hsa-miR-let-7f. The former is known to regulate the expression of BMP2, and consequently the osteogenic differentiation of hBMSC^29^, while the latter was shown to be crucial for the regulation of β-catenin activity and osteogenic differentiation by TIMP-1^30^. For these reasons, it may be interesting to further study the association of these variants with BMD, as well as their functionality.

### Strengths and weaknesses

The resequencing approach in a truncate selection carried out in this study, together with deep *in silico* functional assessment, allowed us to describe potentially relevant common and rare variants present in extreme BMD groups in our cohort. We were able to identify variants that are influencing BMD in the general population, as well as rare variants that may explain extreme phenotypes. This has enabled us to find the rare variants variants rs190011371, rs113001389 and rs570754792, which would have gone unnoticed in association studies. We have performed a comprehensive analysis of the whole coding region, intronic flanking regions, 5′ and 3′UTR and relevant regulatory regions of three important Wnt pathway genes. On the other hand, our approach has two main limitations: the sample size of the study and the lack of analysis of most of the introns and far regulatory regions.

### Final remarks

This work provides variants with indications of functionality in 3 important genes of the Wnt pathway. Specifically, 4 missense variants have been found, three of them nominally associated with BMD. However, the only variants significantly associated were two putative regulatory variants in *WNT16*: rs55710688 (Kozak sequence) and rs142005327 (putative enhancer). In addition, two rare variants in functional regions (*WNT16* in 3′UTR and *SOST* in TATA box) may help determine a low BMD phenotype. Therefore, this work reinforces the importance of regulatory variants in these genes in bone function and opens new ways of research. To confirm our hypotheses, future research may include functional studies to validate the *in silico* predictions of the associated variants and replication of the association studies in different (and preferably larger) cohorts.

## Methods

### Study Cohort

The BARCOS cohort consisted of 1490 postmenopausal women from the Barcelona area, monitored at the Menopausal Unit of the Hospital del Mar (Barcelona, Spain), all of Spanish descent. BMD of all participants was measured at the lumbar spine (LS) and the femoral neck (FN) by dual energy X-ray absorptiometry (DXA). The following data were also recorded: age, age of menarche and menopause, number of fractures and anthropometric measures such as weight and height. DNA is available from all samples of the cohort. Details of the cohort and DNA extraction have been described previously^31,32^.

For the resequencing of the candidate genes, two extreme BMD groups were selected from the BARCOS cohort. For the election of the extreme groups the statistical Z-score was used. The 55 women with the highest Z-score scores (from 2.98 to 0.695) were included in the HBM (High Bone Mass) group and the 53 women with the lowest Z-scores (−2.40 to −4.26) were included in the LBM (Low Bone Mass) group.

Written informed consent was obtained from all patients in accordance with the regulations of the Clinical Research Ethics Committee of Parc de Salut Mar, which approved the study. Samples from patients were obtained in accordance with the Helsinki Declaration, as revised in 2000. All experiments and protocols were approved by the Bioethics Committee of Universitat de Barcelona (IRB00003099).

### Re-sequencing

Resequencing of the *WNT16*, *DKK1* and *SOST* genes was performed in two extreme subgroups of the BARCOS cohort according to the Z-score statistic score. The selection of fragments to be amplified from the *WNT16*, *DKK1* and *SOST* genes and the design of the primers were based on the consensus sequences ENSG00000002745, ENSG00000107984 and ENSG00000167941, respectively (GRCh37.p13, ENSEMBL). *WNT16* was amplified into 6 fragments; *DKK1* in 5 fragments and *SOST* in 7 fragments. The resulting amplicons included all of the coding exons, the UTR regions, the corresponding flanking intronic regions and the two different protein coding transcripts of *WNT16*. Finally, a pair of primers were used for the amplification of the 252 bp of the ECR5 enhancer of *SOST*. Amplification of each of these fragments was done by PCR using Go Taq Flexi DNA polymerase (Promega). The temperature and magnesium conditions of each PCR, and the sequence of the primers are presented in Supplementary Table S5. The PCR fragments were analyzed by agarose gel electrophoresis and purification was done in MultiScreen TM Vacuum Manifold 96-well plates (Merck Millipore). The purified PCR products were sequenced by the Sanger method by the CCiTUB genomic service (Genómica, Parc Cientific, Barcelona, Spain). The tagging kit used is BigDye™ Terminator v3.1 Cycle Sequencing Kit, detection and electrophoresis are performed on automated capillary sequencer models 3730 Genetic Analyzer and 3730xl Genetic Analyzer.

### SNV Genotyping

Genotyping of 13 selected variants in the complete BARCOS cohort was carried out at LGC Genomics (Hoddesdon, UK). In addition, as BARCOS was included in the replication phase of the study by Estrada *et al*.^6^, the genotyping results of SNPs rs3801387 (*WNT16*), rs1373004 (*DKK1*) and rs4792909 (*SOST*) were also available. The genotyping of 12% of the samples was performed in duplicate, as a genotyping quality control, and showed a concordance above 99%.

### Linkage Disequilibrium Calculation

The Haploview software from HapMap (http://hapmap.ncbi.nlm.nih.gov/) was used to calculate and represent the degree of linkage disequilibrium between genotyped polymorphic variants^33^ using the default parameters.

### Haplotype construction and association

Haplotypes of *WNT16* associated SNPs were built using PLINK (http://pngu.mgh.harvard.edu/purcell/plink) with default parameters^34^. Association of haplotypes with LS- or FN-BMD was assessed with the same software. Correction for multiple testing was performed using the Bonferroni’s method. Fisher’s exact test was used to assess the *WNT16* haplotype frequency differences between the two extreme groups of BMD, and between the two halves of the LS-BMD distribution of the complete BARCOS cohort.

### Statistical Analysis

Statistical analyses were performed using Rstudio version 3.3.2. Hardy-Weinberg equilibrium (HWE) was calculated using Chi-square test (X^2^). *p*-values less than 0.01 were considered significant. Fisher’s exact test was used to determine the differences between the two extreme groups of BMD on the genotype frequencies of all detected variants. Linear regression analysis adjusted by years since menopause (YSM), using SNPassoc package, was used to determine the association between BMD and the genotype of each genotype variant. All analyses were performed by testing the additive, the recessive and the dominant model. *p*- values less than 0.05 were considered nominally significant. Correction for multiple testing was performed using the Bonferroni’s method for the number of SNPs tested (n = 13).

### Bioinformatic Analysis and in silico Predictions of the Effect of the Variants

To identify and characterize all variants, we used information from the Ensembl database GRCh37.p13 (http://www.ensembl.org). Information was sought on the MAF of each of the variants found in the European population (EUR) and in the Iberian Peninsula (IBS). We used SIFT (http://sift.bii.a-star.edu.sg/), PolyPhen (http://genetics.bwh.harvard.edu/pph2/) and MutationTaster (http: //www.mutationtaster. org /) to test the effect of the missense variants. MiRNSP (bioinfo.bjmu.edu.cn); miRNA-SNP (www. bioguo.org/miRNASNP); SNP Function Prediction (https://snpinfo.niehs.nih.gov/snpinfo/snpfunc.html); MicroSNiPer (http: // epicenter.ie-freiburg.mpg.de/services/microsniper/); miRdSNP (http://mirdsnp.ccr.buffalo.edu); miRTarBase (http://mirtarbase.mbc.nctu.edu.tw/) were used to analyze the binding of miRNA to the variants in the 3′UTR region. The GTEx database (www.gtexportal.org/home/) was used to identify variants that act as eQTLs. Human Splicing Finder (umd.be) and SplicePort (http://spliceport.cbcb.umb.edu/) were used to evaluate the predicted splicing sites. To characterize regions that include the variants found, the UCSC Genome Browser GRCh37/hg19 was enriched with ENCODE data from osteoblasts.

## Electronic supplementary material

Supplementary Material
