# Supplementary material for: Common and rare variants of WNT16, DKK1 and SOST and their relationship with bone mineral density
Source: Sci Rep. 2018 Jul 19;8:10951. doi: 10.1038/s41598-018-29242-8 (PMC6053384; doi:10.1038/s41598-018-29242-8)
Supplement: Supplementary file 1 — Supplementary Material [file 41598_2018_29242_MOESM1_ESM.pdf]

# Common and rare variants of *WNT16*, *DKK1* and *SOST* and their relationship with bone mineral density

Núria Martínez-Gil[1], Neus Roca-Ayats[1], Anna Monistrol-Mula[1], Natàlia García-Giralt[2], Adolfo Díez-Pérez[2], Xavier Nogués[2], Leonardo Mellibovsky[2], Daniel Grinberg[1#], Susana Balcells[1#*]

[1]Department of Genetics, Microbiology and Statistics, Faculty of Biology, University of Barcelona, IBUB, IRSJD, CIBERER, Barcelona, Spain

[2]Musculoskeletal Research Group, IMIM (Hospital del Mar Medical Research Institute), Centro de Investigación Biomédica en Red de Fragilidad y Envejecimiento Saludable (CIBERFES), ISCIII, Barcelona, Spain

**Supplementary Table S1.** Variants that affect the binding of miRNA at least in 3 different miRNA databases (MiRNSP; miRNA-SNP; SNP Function Prediction; MicroSNiPer; miRdSNP; miRTarBase).

| Gene | Variant | miRNA | Gain/loss | Log FC # | Adj. p-val # |
|---|---|---|---|---|---|
| WNT16 | rs17143305 | hsa-miR-541 | Gain | * | Ns |
| | | hsa-mir4263 | Gain | * | Ns |
| | rs190011371 | hsa-miR-383 | Loss | -0.527 | **0.00408** |
| SOST | rs17883310 | hsa-miR-1915-3p | Loss | 1.58 | **0.00104** |
| | rs17886183 | hsa-miR-5583 | Gain | * | Ns |
| | rs75901553 | hsa-miR-98-5p | Loss | * | Ns |
| | | hsa-miR-3190 | Gain | * | Ns |
| | | hsa-miR-let-7a (and b.c.d.f.g) | Loss | -1.819 | **0.007** |

Gain: Minor allele creates a new binding site for the miRNA
Loss: Minor allele abolish the binding site for the miRNA
# Data from De Ugarte et al. (2015)[1]. Data not shown in the publication.
logFC: Logarithm (Osteoporotic/Non-osteoporotic)
* logFC < |0.5|; Ns < 0.05

**Supplementary Table S2.** GTEx eQTL data for all the SNVs found in the resequencing of *WNT16*, *DKK1* and *SOST*

| SNP | Gene | p value | NES | Tissue |
|---|---|---|---|---|
| *WNT16* | | | | |
| rs17143281 | *FAM3C* | 1.3e-11 | 0.65 | Muscle - Skeletal |
| rs55710688 | *FAM3C* | 0.0000020 | 0.20 | Skin - Not Sun Exposed (Suprapubic) |
| | | 0.000016 | 0.18 | Skin - Sun Exposed (Lower leg) |
| | ***WNT16*** | 0.000019 | 0.29 | Adipose - Subcutaneous |
| rs2908004 | *CPED1* | 0.000029 | 0.097 | Artery - Tibial |
| | *FAM3C* | 0.000086 | -0.15 | Skin - Sun Exposed (Lower leg) |
| rs142005327 | *FAM3C* | 5.9e-8 | 0.22 | Skin - Not Sun Exposed (Suprapubic) |
| | | 9.5e-7 | 0.20 | Skin - Sun Exposed (Lower leg) |
| | | 0.000032 | 0.13 | Nerve - Tibial |
| | *CPED1* | 0.000029 | 0.11 | Artery - Tibial |
| rs2707466 | *FAM3C* | 0.000050 | -0.15 | Skin - Sun Exposed (Lower leg) |
| | *CPED1* | 0.000058 | 0.094 | Artery - Tibial |
| rs17143305 | *RP11-3L10.1* | 7.5e-14 | 0.87 | Testis |
| | *FAM3C* | 2.9e-29 | -0.52 | Skin - Sun Exposed (Lower leg) |
| | | 5.9e-26 | -0.51 | Skin - Not Sun Exposed (Suprapubic) |
| | | 2.7e-8 | -0.21 | Nerve - Tibial |
| | | 4.6e-8 | -0.22 | Thyroid |
| | | 7.8e-8 | -0.27 | Stomach |
| | | 8.8e-8 | -0.34 | Esophagus - Mucosa |
| | | 1.0e-7 | -0.42 | Adrenal Gland |
| | | 1.0e-7 | -0.29 | Muscle - Skeletal |
| | | 1.1e-7 | -0.77 | Brain - Spinal cord (cervical c-1) |
| | | 4.8e-7 | -0.20 | Adipose - Subcutaneous |
| | | 9.5e-7 | -0.31 | Brain - Anterior cingulate cortex (BA24) |
| | | 0.0000028 | -0.35 | Pancreas |
| | | 0.0000031 | -0.21 | Adipose - Visceral (Omentum) |
| | | 0.0000032 | -0.24 | Breast - Mammary Tissue |
| | | 0.0000058 | -0.17 | Artery - Tibial |
| | | 0.0000087 | -0.18 | Esophagus - Muscularis |
| | | 0.000022 | -0.28 | Brain - Frontal Cortex (BA9) |
| | | 0.000027 | -0.30 | Brain - Cortex |
| *DKK1* | | | | |
| rs41281546 | ***DKK1*** | 8.6e-7 | -0.29 | Cells - Transformed fibroblasts |
| rs1569198 | *PRKG1-AS1* | 0.000047 | 0.16 | Cells - Transformed fibroblasts |
| rs74711339 | ***DKK1*** | 1.3e-7 | -0.35 | Cells - Transformed fibroblasts |
| *SOST* | | | | |
| rs1237278 | ***SOST*** | 8.9e-18 | -0.26 | Artery - Tibial |
| | | 3.6e-9 | -0.61 | Brain - Cortex |
| | | 5.4e-9 | -0.27 | Artery - Aorta |
| | | 1.1e-7 | -0.43 | Artery - Coronary |
| | | 0.000024 | -0.28 | Heart - Atrial Appendage |
| | *MPP3* | 0.000013 | 0.22 | Thyroid |
| | *MPP2* | 0.000063 | -0.17 | Cells - Transformed fibroblasts |
| rs851058 | ***SOST*** | 3.4e-20 | -0.28 | Artery - Tibial |
| | | 1.1e-10 | -0.29 | Artery - Aorta |
| | | 1.2e-8 | -0.47 | Artery - Coronary |
| | | 4.2e-7 | -0.35 | Heart - Atrial Appendage |
| | *DUSP3* | 0.000069 | 0.10 | Cells - Transformed fibroblasts |
| rs2023794 | *DUSP3* | 6.0e-7 | 0.29 | Cells - Transformed fibroblasts |
| | *MPP2* | 0.000022 | 0.46 | Skin - Not Sun Exposed (Suprapubic) |
| | | 0.000048 | 0.50 | Skin - Sun Exposed (Lower leg) |

NES: Normalized Effect Size

**Supplementary Table S3.** Minor allele frequency (MAF) from 1000 genomes project: total population (ALL), european population (EUR) and Iberian population in Spain (IBS) and the complete BARCOS cohort frequency.

| SNP | Minor allele | ALL | EUR | IBS | BARCOS cohort |
|---|---|---|---|---|---|
| ***WNT16*** | | | | | |
| rs55710688 | CCCA | 0.252 | 0.233 | 0.276 | 0.257 |
| rs2908004 | A | 0.510 | 0.443 | 0.416 | 0.445 |
| rs142005327 | CT | 0.257 | 0.254 | 0.29 | 0.28 |
| rs113001389 | A | 0.004 | 0 | 0 | 0.0007 |
| rs2707466 | T | 0.503 | 0.440 | 0.407 | 0.463 |
| rs190011371 | C | 0.001 | 0 | 0 | 0.002 |
| ***DKK1*** | | | | | |
| rs1569198 | A | 0.682 | 0.507 | 0.477 | 0.468 |
| rs74711339 | G | 0.019 | 0.043 | 0.033 | 0.057 |
| ***SOST*** | | | | | |
| rs1237278 | C | 0.436 | 0.355 | 0.425 | 0.392 |
| rs2023794 | C | 0.074 | 0.044 | 0.037 | 0.056 |
| rs570754792 | A | 0.0004 | 0.002 | 0.005 | 0.001 |
| rs17882143 | T | 0.007 | 0.018 | 0.033 | 0.023 |
| rs17883310 | A | 0.009 | 0.013 | 0.023 | 0.018 |

**Supplementary Table S4.** Effect size of the associated variants

| SNP | Position | Type | Effect size (β and OR) | |
|---|---|---|---|---|
| | | | LS-BMD | FN-BMD |
| *WNT16* | | | | |
| rs55710688 | p.Met1?<br>g.120965467_120965470dupCCCA | Fs<br>5'UP | 0.018 (0.006, 0.031)<br>1.019 (1.006, 1.031) | 0.009 (0.0001, 0.019)<br>1.01 ( 1.0001, 1.019) |
| rs2908004 | p.G72R/p.G82R | M | 0.012 (0.0015, 0.023)<br>1.012 (1.001, 1.023) | |
| rs142005327 | c.346+103_104dupCT<br>c.316+103_104dupCT | I | 0.018 (0.006, 0.03)<br>1.018 (1.006, 1.031) | 0.011 (0.002, 0.02)<br>1.011 (1.002,1.02) |
| rs2707466 | p.T253I/p.T263I | M | 0.014 (0.002, 0.026)<br>1.014 ( 1.002, 1.026) | 0.01 (0.002, 0.019)<br>1.01 (1.002,1.019) |
| rs3801387# | c.603+2747A>G<br>c.633+2747A>G | I | 0.017 (0.005, 0.03)<br>1.017 (1.005, 1.03) | 0.01 (0.0004, 0.019)<br>1.01 (1.0004, 1.019) |
| *DKK1* | | | | |
| rs1569198 | c.548-43A>G | I | | 0.011 (0.003, 0.019)<br>1.011 (1.003,1.019) |
| *SOST* | | | | |
| rs17882143 | p.Val10Ile | M | *0.041 (0.003, 0.079)*<br>*1.042 (1.003, 1.082)* | |

Underline and Italics β and OR under dominant model

# SNP genotyping previously in BARCOS (Estrada *et al*. (2012)[2]).

**Supplementary Table S5.** PCR condicitons. Sequence of the primers (5'→3'), annealing temperature (ºC), amount of magnesium (mM) and elongation time at 72ºC (seconds).

| Primers | Sequence (5'→3') | Tm | $Mg^{2+}$ (mM) | Elongation time (s) |
|---|---|---|---|---|
| WNT16-Frag1a-F<br>WNT16-Frag1a-R | GGTAGCTCCAGTAAGAGATTC<br>CAGATTACCGTGTCTTTGGGT | 62 | 2 | 15 |
| WNT16-Frag1b-F<br>WNT16-Frag1b-R | CGGAGCCGCTCTCCACCA<br>ATTAGGTCACTCGTCTAAGGG | 62 | 2 | 15 |
| WNT16-Frag2-F<br>WNT16-Frag2-R | ACTTTCAACTGAGGCTGGGG<br>CTGGAACTGGGGAGTCAGG | 62 | 2 | 15 |
| WNT16-Frag3-F<br>WNT16-Frag3-R | CTTCCTTTCTAAATATGTACTCG<br>AGGGCTGCCAGTGTTTGGTT | 58 | 2 | 15 |
| WNT16-Frag4-F1<br>WNT16-Frag4-R1 | TGGGACAAAAACCAAAGGACG<br>TGACCACATGGGTGTTGTAAC | 62 | 2 | 15 |
| WNT16-Frag4-F2<br>WNT16-Frag4-R2 | AGGATGATCTGCTCTATGTTAAT<br>CCCACCATTATTGAGTCCTGT | 62 | 2 | 15 |
| DKK1-Frag1-F<br>DKK1-Frag1-R | GCGCTGATCACAGTCCTTATC<br>TGCTATAACGCTCGCTGGTA | 63 | 2 | 15 |
| DKK1-Frag2-F<br>DKK1-Frag2-R | GCAGTGGGCAGTAACAGGT<br>TGTATTGAATCATTGAGGGACA | 62 | 2.5 | 35 |
| DKK1-Frag3-F<br>DKK1-Frag3-R | GAGGAAGTTTGGCTTGTGTTT<br>CGAAGGAGAAGACAGTAGGAAA | 62 | 2 | 15 |
| DKK1-Frag4a-F<br>DKK1-Frag4a-R | GAACCACCTTGTCTTCAAAAATG<br>TCCAAGAGATCCTTGCGTTC | 63 | 1.5 | 20 |
| DKK1-Frag4b-F<br>DKK1-Frag4b-R | AGGTGCTGCACTGCCTATTT<br>CCGTATCCTCATTCCAATCAA | 63 | 1.5 | 20 |
| SOST_Frag0a_F<br>SOST_Frag0a_R | CCGAGTTGGGCAGATCACC<br>TTAATGCAGACGGTCCAGCC | 62 | 1.5 | 15 |
| SOST_Frag0b_F<br>SOST_Frag0b_R | ACGCGTTCCAGGGATGAATC<br>GGCCAAGGCAGCATTTTCTC | 62 | 1.5 | 15 |
| SOST_Frag1_F<br>SOST_Frag1_R | GCTAGAGGAGAAGTCTTGG<br>CCATTCTTCCCCACCTCC | 62 | 2 | 10 |
| SOST_Frag2a_F<br>SOST_Frag2a_R | AGATGTTCAGGGGCAAAAGC<br>GGAAGTCGGGCCCACTAG | 63 | 2 | 30 |
| SOST_Frag2b_F<br>SOST_Frag2b_R | GTCACCGAGCTGGTGTGCT<br>CTCAGGGCCTGGAAGGTCT | 63 | 2 | 30 |
| SOST_Frag3a_F<br>SOST_Frag3a_R | CTCAAGGACTTCGGGACCGA<br>AAATGAGGGTGGAGGTGG | 62 | 2 | 15 |
| SOST_Frag3b_F<br>SOST_Frag3b_R | AGTCCTGGCTCTGCCACTAA<br>GGACACATTTCTGCCTAGAAAA | 60 | 2 | 15 |
| SOST_Frag3c_F<br>SOST_Frag3c_R | GGGGGAAAAACTACAAGTGC<br>TCCTTTCCAAACCCAGACC | 62 | 2 | 15 |
| ECR5_F<br>ECR5_R | TCCTTGCCACGGGCCACCAGCTTT<br>CCCCCTCATGGCTGGTCTCATTTG | 62 | 2 | 5 |

## *WNT16*

rs4727920; g.120965562A>G; c.65+28A>G (WNT16a) / g.120965562A>G (WNT16b); example of one HBM woman carrying the variant.

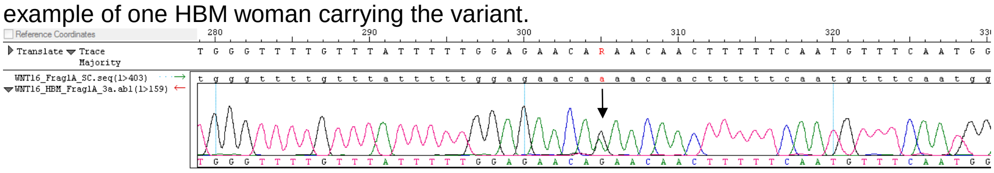

rs201022838; g.120969332C>A; c.66-289C>A (WNT16a) / c.-15C>A (WNT16b); example of the only HBM woman carrying the variant.

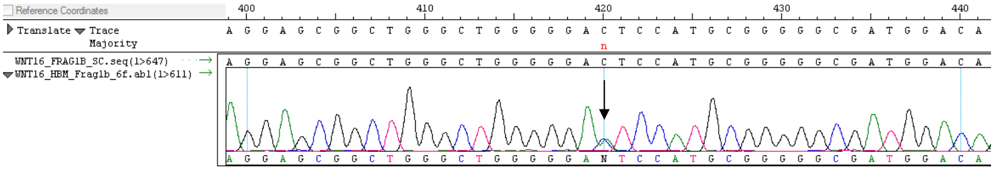

rs17143291; g.120969825C>A; p.Thr90Thr (WNT16a) / p.Thr100Thr (WNT16b); example of one LBM woman carrying the variant.

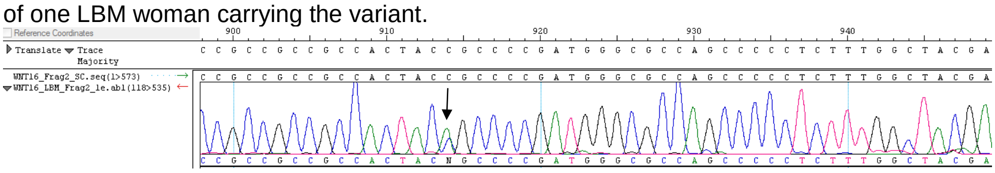

rs147496912; g.120969929G>T; c.316+58G>T (WNT16a)/ c.346+58G>T (WNT16b); example of one HBM woman carrying the variant.

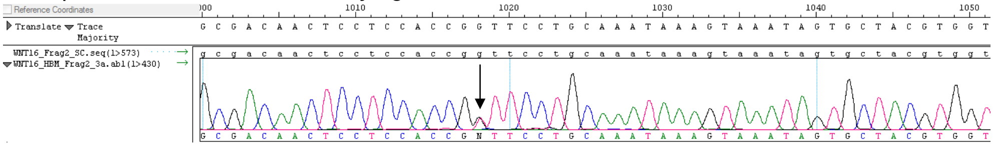

rs140239870; g.120970018A>G; c.316+147A>G (WNT16a)/ c.346+147A>G (WNT16b); example of one LBM woman carrying the variant.

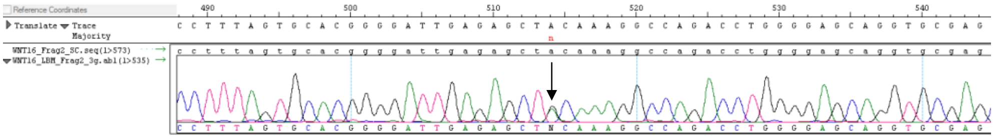

rs113001389; g.120970045C>A; c.316+174C>A (WNT16a)/ c.346+174C>A (WNT16b); example of the only LBM woman carrying the variant.

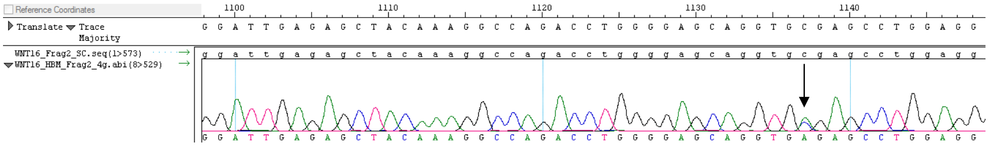

rs190011371; g.120979568G>C; g.120979568G>C (WNT16a) /c.*169G>C (WNT16b); example of the only LBM woman carrying the variant.

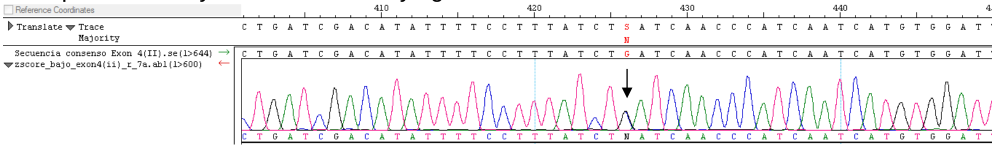

**Supplementary Figure S1.** Examples of DNA sequence chromatograms from various individuals, each containing one of the *WNT16* rare variants described in this paper. In all cases, the variants are in heterozigosis and are signaled by arrows.

## *DKK1*

rs540255939; g.54074079C>A; c.-116C>A; example of the only LBM woman carrying the variant.

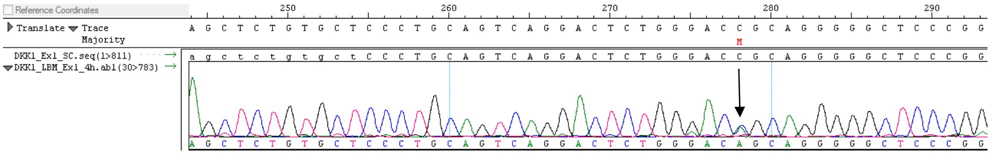

rs149268042; g.54074798G>T; p.Arg120Leu; example of the only HBM woman carrying the variant.

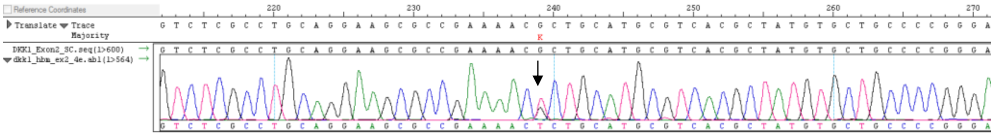

c.406+195G>A; g.54075040G>A; example of the only HBM woman carrying the variant.

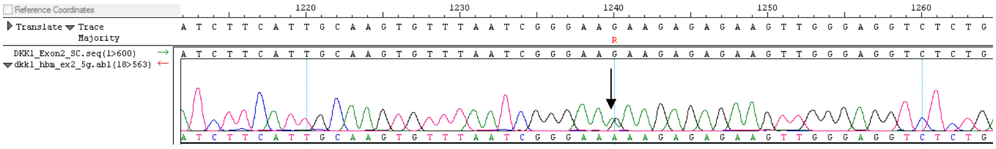

rs200054686; g.54076944delT; c.*377delT, example of the only LBM woman carrying the variant.

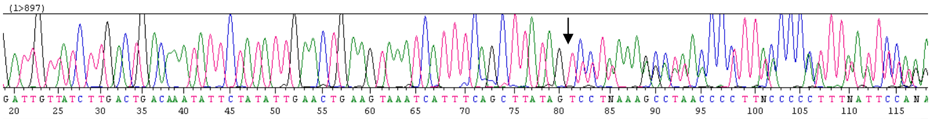

rs953208416; g.54077319C>T; c.*752C>T, example of the only HBM woman carrying the variant.

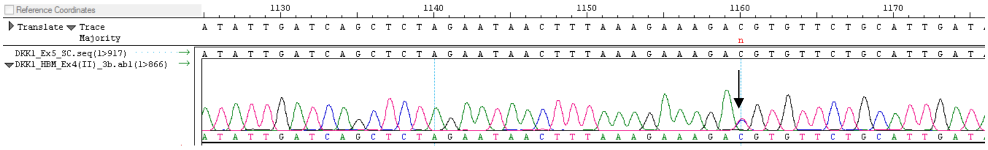

rs79759877; g.54077322G>A; c.*755G>A; example of the only HBM woman carrying the variant.

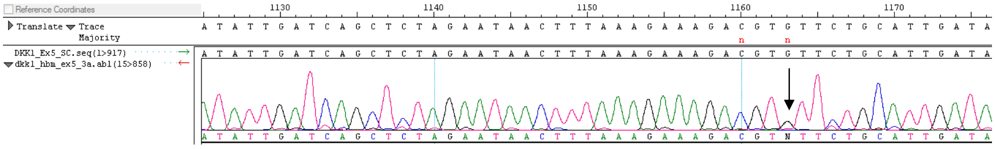

rs549135224; g.54077585G>A; example of one HBM woman carrying the variant.

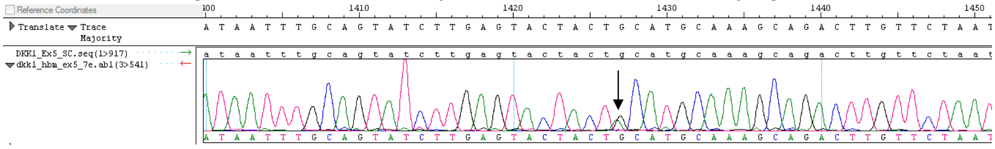

**Supplementary Figure S2.** Examples of DNA sequence chromatograms from various individuals, each containing one of the *DKK1* rare variants described in this paper. In all cases, the variants are in heterozigosis and are signaled by arrows. In the case of rs20054686, which is a heterozygous deletion, the whole chromatogram to the right of it is affected.

## *SOST*

rs184269196; g.41838340G>A; example of one HBM woman carrying the variant.

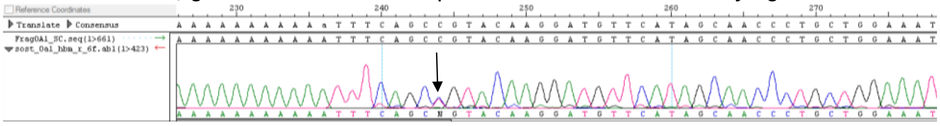

rs79715828; g.41838130G>A ; example of the only HBM woman carrying the variant.

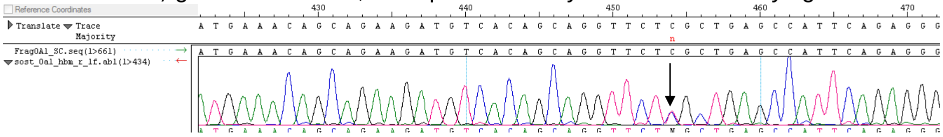

rs74252774; g.41838012G>T; example of the only HBM woman carrying the variant.

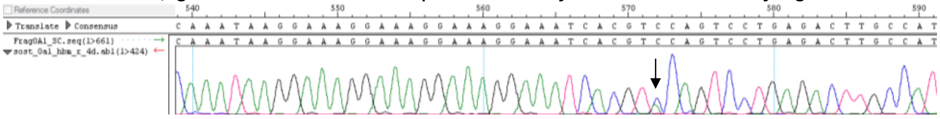

rs567865956; g.41837786C>T; example of one HBM woman carrying the variant.

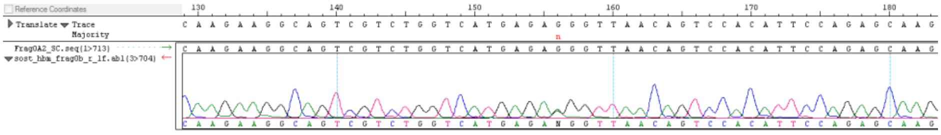

rs61105240; g.41837720C>T; example of the only HBM woman carrying the variant.

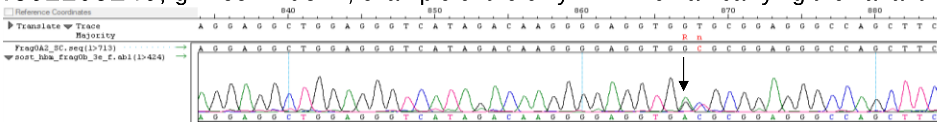

rs115185703; g.41837530C>A; example of the only HBM woman carrying the variant.

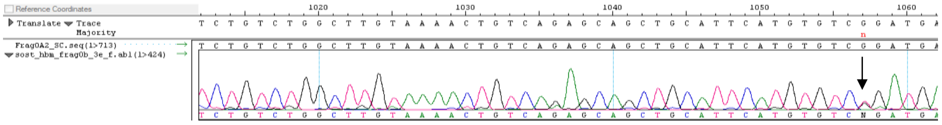

rs570754792; g.41836179G>A; example of the only LBM woman carrying the variant.

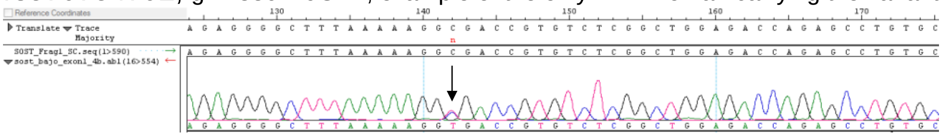

rs17886183; g.41831706C>T; example of one HBM woman carrying the variant.

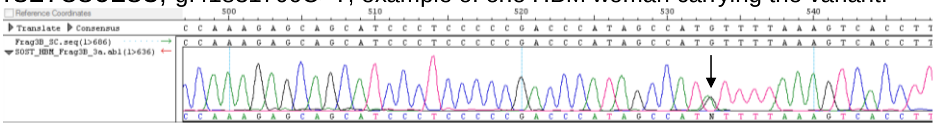

rs17885979; g.41831307delC; c.*1404delG; example of the only HBM woman carrying the variant.

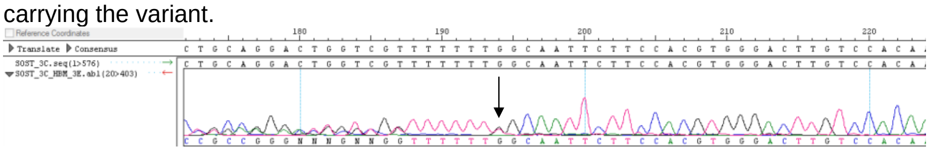

rs566556646; g.41831153C>G; c.*1557G>C; example of the only HBM woman carrying the variant.

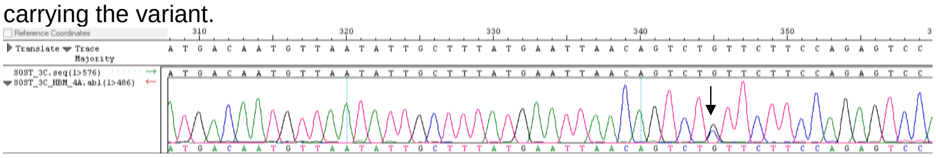

**Supplementary Figure S3.** Examples of DNA sequence chromatograms from various individuals, each containing one of the *SOST* rare variants described in this paper. In all cases, the variants are in heterozigosis and are signaled by arrows. In the case of rs17885979, which is a heterozygous deletion, the whole chromatogram to the right of it is affected.

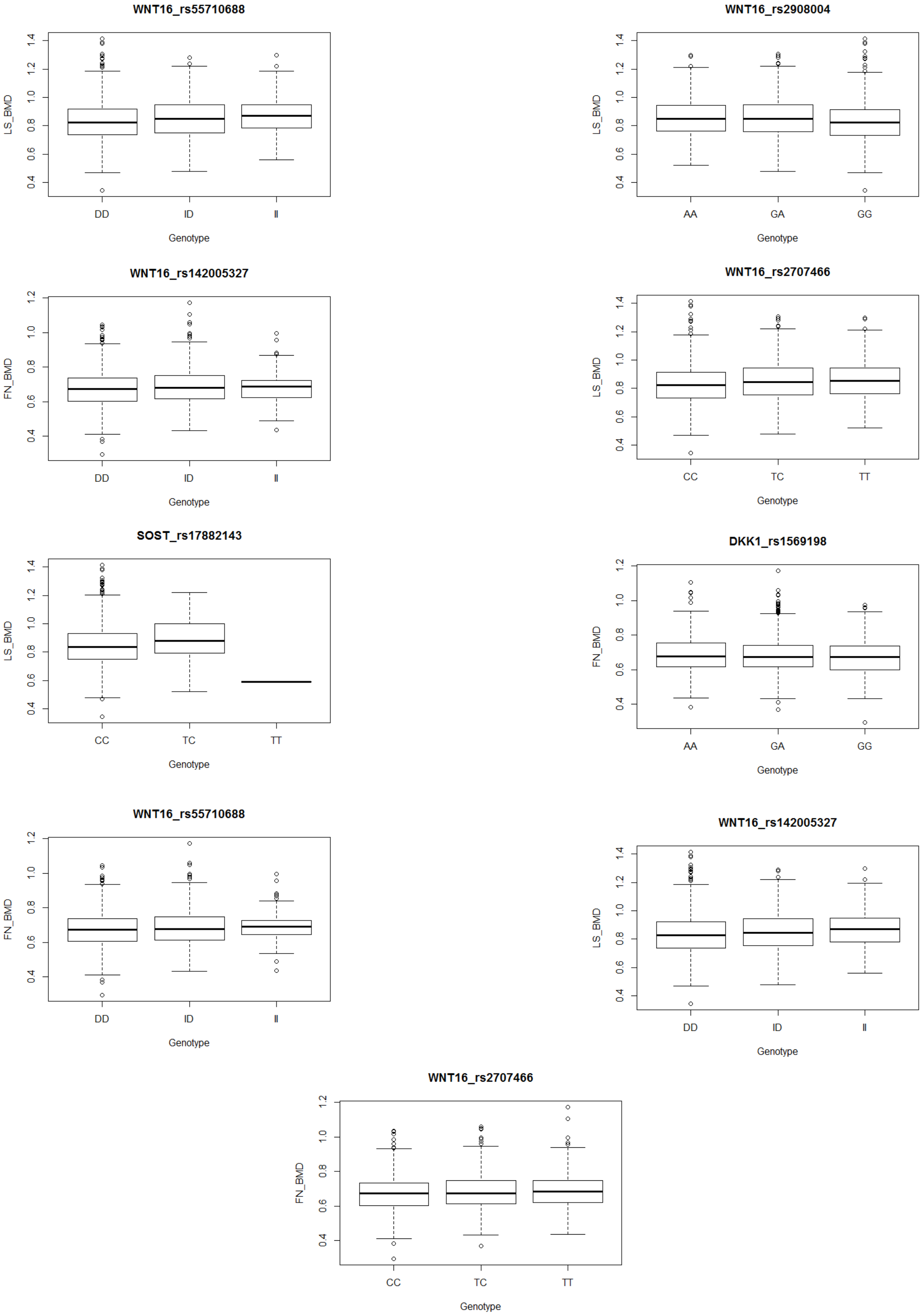


**Supplementary Figure S4.** Boxplots for the nominally significant associated SNPs rs55710688, rs2908004, rs142005327, rs2707466, rs17882143, rs1569198 with LS-BMD or FN-BMD.

# References


1. De-Ugarte, L. et al. MiRNA profiling of whole trabecular bone: identification of ostoeporosis-related changes in MiRNAs in human hip bones. BMC Med. Gen. 8:75 (2015)
2. Estrada, K. *et al.* Genome-wide meta-analysis identifies 56 bone mineral density loci and reveals 14 loci associated with risk of fracture. *Nat. Genet.* **44,** 491–501 (2012).
